# Barriers and facilitators to infection prevention practices in home healthcare: a scoping review and proposed implementation framework

**DOI:** 10.1016/j.infpip.2024.100342

**Published:** 2024-01-30

**Authors:** Lisa Brockhaus, Nikita Sass, Niklaus D. Labhardt

**Affiliations:** Division of Clinical Epidemiology, Department of Clinical Research, University Hospital Basel and University of Basel, Basel, Switzerland

**Keywords:** Infection prevention and control, Home healthcare, Home environment, Standard precautions, Healthcare providers

## Abstract

Infection prevention and control (IPC) research has focused on the hospital setting, neglecting the rapidly expanding home healthcare (HHC) sector. Current infection prevention recommendations do not reflect the challenges specific to the HHC setting.

This scoping review considered any original studies reporting on barriers or facilitators to infection prevention practices in the context of HHC. Study characteristics were mapped, and a descriptive content analysis was performed. Based on the findings we propose a framework of eight HHC setting characteristics relevant to infection prevention implementation.

33 studies fulfilled the eligibility criteria. A majority of studies addressed sharps injury or blood and body fluid exposure prevention (*N*=15) and the majority were conducted in the United States (*N*=23). Study methodologies employed were surveys (*N*=18), qualitative (*N*=11), direct observation (*N*=7), and one interventional study. The HHC setting characteristics relevant to infection prevention implementation were: the care process in the patient's immediate environment; the need to bring equipment and materials into the home; the provision and financing of equipment and materials; the use of patient space and facilities; the unique position of and the expectations towards HHC providers; working alone with little support; the intermittent nature of care; the attitudes of HHC providers formed by their work circumstances.

Interventional studies generating higher-quality evidence for implementation are lacking. Furthermore, implementation of aseptic technique and the decontamination and reprocessing of equipment are poorly studied in the HHC setting and deserve more research interest. The proposed framework may guide future research and implementation work.

## Introduction

The home healthcare (HHC) sector is rapidly expanding in many high-income countries, reflecting ageing populations, and shifts from institutional to home-based care [[1], [2], [3]]. Structural and financial pressures resulting in earlier hospital discharges, and developments in technology-enabled care, further contribute to increasing complexity and severity level of patients being cared for at home [[4], [5], [6], [7]].

Infection prevention and control (IPC) research and quality improvement initiatives have focused on the more structured hospital setting, and neglected the heterogeneous HHC sector [8,9]. The Covid-19 pandemic, however, has raised the awareness that hospital IPC recommendations cannot be easily transferred to care which is delivered in the less controlled environment of the patient's own home. The lack of setting-specific guidance was perceived as a major challenge by HHC organisations during the COVID-19 pandemic [10,11].

The burden of healthcare-associated infections (HAI) reported in HHC patients varies widely between studies, with a systematic review in 2014 reporting infection rates of 4.5–11.5% [12]. True infection rates may vary according to context and may potentially be underreported.

The World Health Organization (WHO) has defined a set of minimum standard infection prevention and control (IPC) practices aiming to protect both healthcare workers and patients by reducing the risk of transmission of microorganisms. These practices, referred to as *standard precautions*, “should be used by all healthcare workers, during the care of all patients […], in all settings” [13]. Key elements of standard precautions include: (1) risk assessment; (2) hand hygiene; (3) respiratory hygiene; (4) personal protective equipment; (5) aseptic technique; (6) safe injections and sharps injury prevention; (7) environmental cleaning; (8) waste management; (9) decontamination and reprocessing of reusable patient care items and equipment [13]. A variety of WHO implementation tools are available to healthcare facilities for the implementation of IPC. However, none of them addresses the specific context and challenges of IPC in home healthcare [14,15].

Studies suggest that in the HHC setting, compliance of healthcare workers (HCWs) with infection prevention guidelines is low. The average adherence rate to hand hygiene opportunities was 46% in a recent study conducted in the United States (US) [16]. A Belgian study reported compliance to three care bundles to prevent central-line associated bloodstream infections (CLABSI) between 0% and 22% [17]. However, the respective guidelines, such as the WHO Five Moments for Hand Hygiene [18], or prevention bundles for CLABSI [19], are tailored to hospital settings.

## Rationale, research questions and objectives

A scientific debate or consensus about the rationale for, and nature of, necessary adaptions of hospital-derived IPC recommendations to the HHC setting is widely lacking [8,20]. A limited set of studies has explored the challenges to IPC in HHC, using a variety of methodological approaches, and addressing varying infection prevention practices. Using a scoping review methodology, we aimed to explore the breadth and depth of the available evidence.

A search of MEDLINE, JBI Evidence Synthesis, the Cochrane database (Cochrane reviews and Cochrane protocols) and PROSPERO did not yield any recent or ongoing review activity to our review questions.

This review addresses the following research questions:

• What are the barriers to infection prevention practices encountered by HHC practitioners regarding care procedures in the patient's home?
• What are the strategies HHC practitioners use to mitigate these barriers?

The objectives of this review were to:

(1) Map the research landscape of studies addressing barriers or facilitators of infection prevention practices in HHC, regarding their focus of interest and the methodologies used.
(2) Provide a descriptive content analysis of the study findings.
(3) Based on the findings, develop a framework of HHC setting characteristics challenging infection prevention to guide future research and implementation work.
(4) Critically discuss the study landscape regarding limitations and knowledge gaps.

## Methods

The methodology of this scoping review was based on the Joanna Briggs Institute Manual for Evidence Synthesis [21], with the PRISMA extension for Scoping Reviews [22] as a reporting guideline, and the PRISMA 2020 flow diagram for systematic reviews [23] to depict the study selection process. The protocol was registered in OSF (https://www.cos.io/products/osf) registries on 13 Feb 2023 [24].

### Ethics statement

Ethical approval was not required, the study exclusively used data in the public domain.

### Information sources

Searches were performed in MEDLINE (via Ovid), CINAHL (via EBSCO), and EMBASE (via Ovid). No language or date limits were applied. Articles in languages other than English were considered according to translation resources within the wider study team.

### Search strategy

The search strategy was iteratively developed from an initial limited search, with analysis of the keywords and index terms used in the retrieved articles. The search string was developed in MEDLINE and refined with the input of an experienced medical information librarian. Keywords were then transcribed to the remaining databases using the Systematic Review Accelerator Polyglot search tool [25], reviewed and edited as needed. Subject headings were manually transcribed. The full search strings are available in Appendix A. The search was run on 7 February 2023, and updated on 17 April 2023.

### Study selection process

The study selection process consisted of three stages: (1) a title- and abstract screening of the identified publications for potential eligibility; (2) a full-text screening evaluating the selected publications against all inclusion/exclusion criteria; and (3) a hand search of the reference lists of the included publications. The de-duplication and selection process was performed in Covidence (www.covidence.org).

A random sample of retrieved articles was piloted against the inclusion and exclusion criteria by both reviewers to refine the eligibility criteria. The three stages were then performed by two reviewers independently. Any discordances were resolved by discussion, or, if necessary, with a third reviewer. Authors of potentially eligible conference abstracts were contacted for further detail if no corresponding full-text publication was identified.

### Eligibility criteria

*Participants:* This review considered studies that included any professional providers of HHC. Those include practitioners of any professional training level (such as nurses, physicians, home health aides), and agency executives in varying roles (such as infection preventionists). We excluded studies researching perspectives of informal caregivers such as family members, and patients.

*Concept:* Studies that explored or evaluated factors associated with the execution of IPC practices in HHC were considered. Studies were included if at least one of the objectives referred to in the research questions or, if objectives were not stated, a substantial part of the result section.

Factors associated with the execution of IPC practices were classified as barriers (i.e. any factor negatively impacting), or facilitators, or mitigation strategies (i.e. any factor perceived to be positively impacting).

Infection prevention practices were any of the key elements of WHO standard precautions [13], or infection prevention precautions in a generic sense. Studies exclusively about factors associated with IPC policies, agency-level infrastructure, or describing adherence rates to infection prevention practices, were excluded. The link to infection prevention practices had to be apparent in the manuscript. Studies focusing on factors that may suggest a link to infection prevention, but that were not evaluated in that context by the authors, were excluded.

*Context:* This review was exclusively interested in provision of healthcare in the home environment. Healthcare for this review included medical care, specialised or qualified nursing care, and basic healthcare services such as assistance with activities of daily living.

Studies focusing on household help were excluded. Studies addressing a variety of settings including the home environment, were included if they stratified results according to the care setting, if such sub-data were available from the authors, or if the study concepts closely related to the home environment. Studies conducted in low- and lower-middle-income countries, as per World Bank country classification 2023 [26], were excluded.

*Types of sources:* This review included any type of primary study design case series, and case studies. Guidance documents, viewpoints, and studies with insufficient detail regarding the research questions were excluded.

Minor refinements of the eligibility criteria were made during the study selection process, in line with the suggested iterative approach of a scoping review [27]. A protocol amendment was agreed on within the study team to include observer-perceived factors as a concept, in addition to HCW-perceived factors. Participant observation studies (that is, the observation of HCWs by an external observer, referred to as direct observation studies in this review) provided data relevant to the research questions.

### Data extraction

A data extraction tool was customised using Covidence software (www.covidence.org) and piloted on four studies by both reviewers. The following study characteristics were extracted: First author; publication year; year of study conduct; type of journal; country; study objectives that relate to the research questions; methodology; intervention type/comparator, participant characteristics; number of participants (and, for survey studies, response rate); findings that relate to the research questions; infection prevention practice(s) of interest; study limitations as identified by the authors; specific context as discussed by the authors.

For multi-method studies addressing more than one research question, only the methodological approaches that addressed our research question were extracted as study methodology of interest (e.g., in a study using focus groups for healthcare workers, and interviews for patients, only the focus groups were extracted).

The infection practice of interest was selected from the WHO set of standard precautions, or, if not specified by the study, classified as “IP general”. Studies were classified to more than one practice of interest if those were explicitly stated in the publication.

Data extraction was performed by the two reviewers independently and discrepancies resolved by discussion.

### Data analysis

Descriptive statistics of study characteristics were performed in R, Version 4.2.2. (www.r-project.org). Where several publications originated from the same study dataset, they were analysed separately if they addressed different research questions.

A descriptive qualitative content analysis of the study findings was performed as outlined by the Joanna Briggs Institute [28], using Microsoft ® Excel ®. Study findings were open-coded and deductively allocated to the corresponding infection prevention IPC practice as initial categories. Findings overarching more than one infection prevention practice, or being of general nature, were summarised in the “IP general” category. Categories with very limited findings were merged with the most related practices. If studies listed findings as both barriers, and the opposite finding as facilitators, the facilitator was not listed. Findings were then further organised according to the PETT (people – environment – tools – tasks) scan tool that aligned well with the study findings. This tool, developed by the Systems Engineering Initiative for Patient Safety (SEIPS), has been used previously to describe barriers and facilitators within complex work systems in healthcare settings [29].

Based on the findings organised as described, an inductive approach was used to develop the implementation framework of HHC setting characteristics. Findings not reasonably specific to the HHC setting were not considered in this framework.

## Results

### Study inclusion

A total of 2663 abstracts were screened, with 82 undergoing full-text review. 33 publications were included. One article was not assessed at full-text screening stage for lack of translation resources (Chinese). A flowchart of the study selection process is provided in Appendix B.

### Characteristics of included studies

Table I summarises the characteristics of the included studies. The majority of studies were conducted in the United States (US) (*N*=23), fewer studies in the United Kingdom (UK) (*N*=2), Germany (*N*=2), Belgium (*N*=1), the Netherlands (*N*=1), Australia (*N*=2), and Brazil (*N*=1). 31 of the publications were in English, 2 in German. 3 of the recent studies primarily reflected on the COVID-19 pandemic [[30], [31], [32]]. All included studies were published in peer-reviewed journals. No conference abstract publications fulfilled the selection criteria.

**Table I:** Studies included

Author, year	Country	Methodology	Infection prevention practice
Adams *et al.* [34], 2020	US	Survey	general
Adlers *et al.* [46], 2012	Germany	Survey	general
Amuwo *et al.* [64], 2011	US	Survey	Sharps injury/blood exposure prevention
Amuwo *et al.* [33], 2013	US	Interventional study with comparator	Sharps injury/blood exposure prevention
Backinger *et al.* [65], 1994	US	Survey	Sharps injury/blood exposure prevention
Bennett *et al.* [49], 2004	UK	Survey	Hand hygiene; Sharps injury/blood exposure prevention
Brouillette *et al.* [41], 2017	US	Survey	Sharps injury/blood exposure prevention
CorrêaCordeiro *et al.* [45], 2016	Brazil	Interview study	PPE
Dowding *et al.* [36], 2020	US	Interview study	general
Dowding *et al.* [58], 2020	US	Direct observation study; Interview study	general
Felembam *et al.* [48], 2012	Australia	Direct observation study	Hand hygiene
Felemban *et al.* [38], 2015	Australia	Interview study; Focus groups	general
Gershon *et al.* [50], 2009	US	Survey	Sharps injury/blood exposure prevention
Hallett [52], 2000	UK	Interview study	Aseptic technique
Kenneley [54], 2012	US	Survey	general
Kim *et al.* [47], 2010	US	Survey	Sharps injury/blood exposure prevention
King *et al.* [32], 2022	Canada	Survey	PPE
Leiss [44], 2010	US	Survey	Sharps injury/blood exposure prevention
Leiss *et al.* [53], 2011	US	Survey	PPE
Leiss [56], 2012	US	Survey	Sharps injury/blood exposure prevention
Leiss [55], 2014	US	Survey	PPE; Sharps injury/blood exposure prevention
Markkanen *et al.* [39], 2007	US	Interview study; Focus groups	Sharps injury/blood exposure prevention
Markkanen *et al.* [42], 2015	US	Interview study	Sharps injury/blood exposure prevention
McDonald *et al.* [16], 2021	US	Direct observation study	Hand hygiene
Osakwe *et al.* [30], 2021	US	Interview study	general
Osei-Poku *et al.* [31], 2021	US	Focus groups; Survey	general
Pogorzelska *et al.* [51], 2020	US	Interview study	general
Popp *et al.* [40], 2006	Germany	Direct observation study	general
Quinn *et al.* [57], 2009	US	Survey	Sharps injury/blood exposure prevention
Russell *et al.* [35], 2021	US	Direct observation study; Interview study	general
Sitzman *et al.* [37], 2009	US	Survey	general
Steffens *et al.* [17], 2019	Belgium	Direct observation study	Sharps injury/blood exposure prevention
Wendt *et al.* [43], 2022	Netherlands	Direct observation study; Focus groups	Hand hygiene; PPE; Sharps injury/blood exposure prevention; waste management; decontamination/reprocessing; risk assessment

PPE, personal protective equipment; UK, United Kingdom; US, United States.

A large proportion of studies focused on sharps injury/blood exposure prevention (*N*=15), followed by studies not explicitly listing a practice of interest (*N*=12). Fewer studies focused on personal protective equipment (*N*=5), hand hygiene (*N*=4), aseptic technique, decontamination/reprocessing of reusable equipment and materials, waste management, or risk assessment (*N*=1, respectively).

Methodological approaches employed were qualitative (interview studies and/or focus groups) (*N*=11), a survey design (*N*=18), direct observation (*N*=7), and an intervention study with comparator (*N*=1).

The only intervention study identified aimed to assess the effectiveness of an educational intervention on blood and body fluid exposure among home care aides. The intervention consisted of interactive trainings facilitated by peer educators, and the development of communication tools for patients and professionals [33].

### Descriptive content analysis of study findings

**Figure 1:**
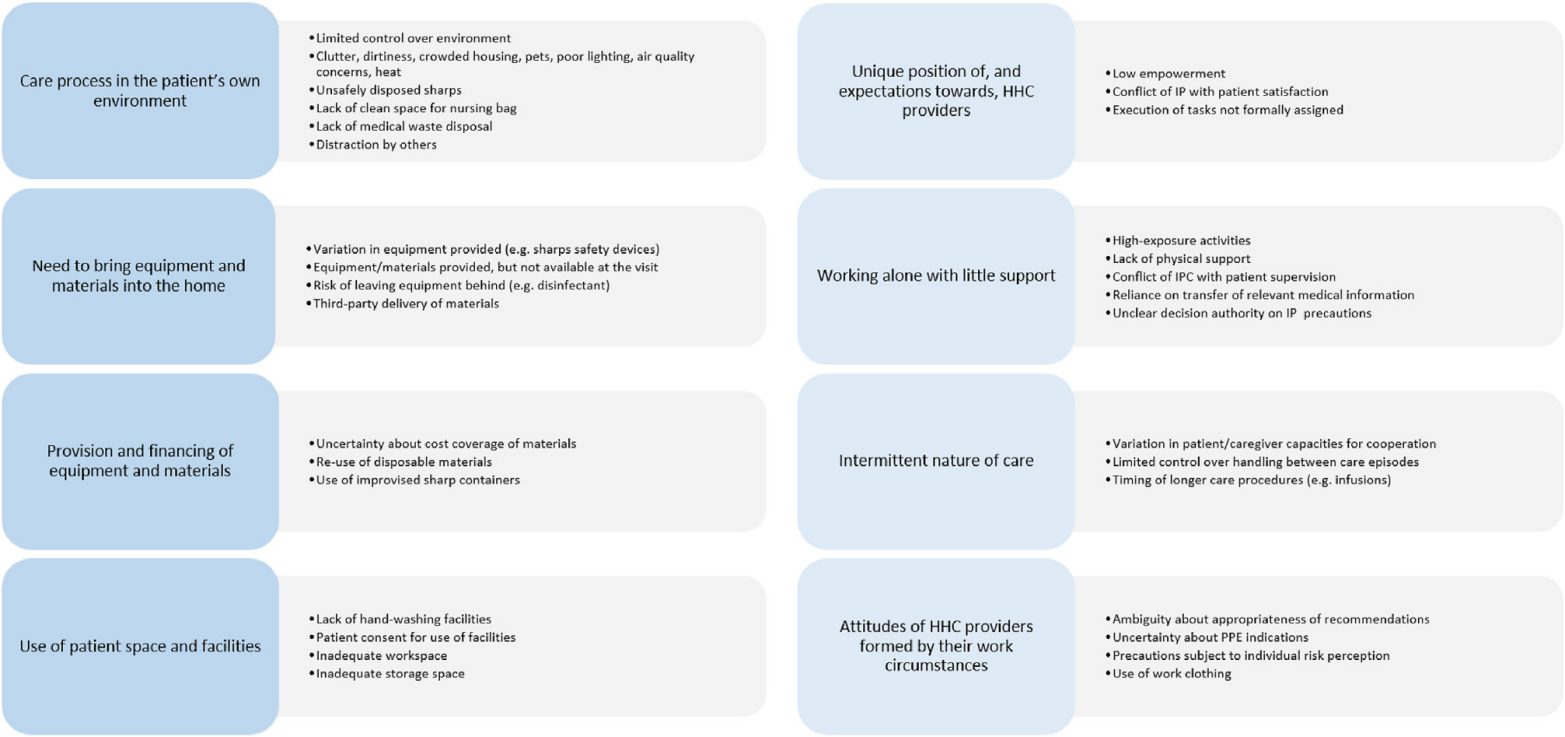
Proposed framework of home healthcare setting characteristics relevant to infection prevention implementation. HH: hand hygiene; HHC: home healthcare; IP: infection prevention; IPC: infection prevention and control; PPE: personal protective equipment.

Table II provides a list of study findings. Figure 1 outlines the proposed framework of overarching HHC setting characteristics challenging the implementation of infection prevention practices:

(1) *The care process taking place in the patient's own environment:* A large number of studies found diverse adverse environmental conditions such as clutter, dirty environment, or crowded housing negatively affecting infection prevention [16,[34], [35], [36], [37], [38], [39], [40]]. Further notable findings, illustrating the limited control over the home environment, included patients leaving unsafely disposed sharps around the house [39,41,42], and distraction by other people being present during the delivery of care [34,37,43].
(2) *The need to bring equipment and materials into the home:* Challenges regarding availability of equipment at the specific visit were not limited to anticipation of equipment needs [44,45]. Studies also revealed challenges of using a variety of equipment from different providers including patient-procured items (such as, sharps safety devices) [39,42], reliance on third-party delivery of materials, and equipment storage by the patient [38,43].
(3) *Provision and financing of equipment and materials:* Several studies noticed that costs for IPC materials, such as PPE, were not re-financed, cost coverage was unclear, or dependent on the patient's insurance [36,38,46]. Financial constraints further led to the use of unsafe improvised sharps containers by patients [44], and re-use of disposable materials after cleaning with hand disinfectant [43].
(4) *Use of patient space and facilities:* Lack of adequate workspace and storage space was a major theme in a large number of studies [17,38,39,43,47]. It was noted that some households lacked clean towels for hand drying [43,48]. Studies further emphasised that patients needed to consent to the use of their facilities such as sinks [49].
(5) *Unique position of and expectations towards HHC providers*: Studies revealed that HHC professionals lacked the power to impose safety practices on patients, or to refuse care if they felt it was unsafe [31,41,43,50]. PPE use had to be negotiated when it was not appreciated by a patient [30,49]. HCWs were reported to feel pressured to execute tasks that were not formally assigned, such as helping the patient with sharps use [33,41].
(6) *Working alone with little support:* Studies revealed impracticality of correct PPE use and hand hygiene adherence during typical tasks, such as helping a patient with mobility, or bathing a patient, which was perceived as a high-exposure-risk activity for HCWs [31,39,47]. HHC professionals were found to rely on the varying quality of medical information (e.g. regarding colonisation with multi-drug resistant organisms (MDRO)) being transferred from other stakeholders [30,36,43,46]. One study described the decision authority on necessary precautions being uncertain, and unsatisfactory if left to physicians [46].
(7) *Intermittent nature of care:* Studies emphasised that HHC providers had limited control over procedures between care episodes [35,36,51], but also had to deal with a varying degree of cooperation capacity of patients or caregivers [36,51]. One study revealed challenges to the aseptic handling of infusions, given that HHC providers could not be present during the whole procedure [40].
(8) *Attitudes of HHC providers formed by their work circumstances:* Studies revealed some ambiguity of HHC professionals regarding the usefulness and feasibility of existing recommendations, such as for the WHO Five Moments for Hand Hygiene [43] or guidance on aseptic technique [52]. Confusion about PPE indications was found to negatively impact infection prevention [52,53]. Adherence to practices was generally found to be subject to individual risk perception [32,53]. Studies further reported overreliance on hand hygiene when handwashing was indicated [51], work clothing not being used as provided or improperly washed [40,43], and smartphones and tablets used at the visit not properly cleaned because of uncertainty about the correct disinfection agent [43].

**Table II:** Descriptive qualitative content analysis of study findings

General	Barriers	Facilitators
People • HHC professionals • Patients • Others	lack of knowledge and insufficient training of nurses [46,51] insufficient training of staff in charge of IPC [51] lack of empowerment of nurses, reliance on trust, impossible to refuse care [31,43] staffing challenges: poor retention and recruitment, having to take on additional responsibilities [51] work clothing provided but not worn/at the discretion of nurses [43] same clothing used off working hours [40] clothing to be washed by nurses, not washed at the recommended temperature [40,43] patient preference as a priority, conflict of IPC with home environment [46] verbal abuse, (threat of) physical assault [41,50] poor patient hygiene [34,35] poor patient knowledge [36,46] cognitive impairment of patient [36] patient and family not following advice, resistance to change [36,51] some patients do not appreciate work clothing [40] no suitable caregiver [36] other people present during delivery of care, unruly children [34,37,43] physicians do not provide indication for IPC measures, decision authority exclusively for physicians [46]	Patient and caregiver education, repetitive education, modelling behavior [35,36,39,51] Assess patient/caregiver capacity for following advice [36] Find a caregiver and provide education to them [35] Agreements with patients [31] Referral to a social worker to assess environment [35] Suggest domestic help or pest control [38]
Environment • Physical • Socio-organisational	limited control over home environment [35] no control over (Covid-19) precautions of patient and family [31] clutter [[34], [35], [36], [37], [38], [39],^59^] dirty environment [34,35,40,43,51,59] insects, rodents, infestations [34,36,39,50]crowded housing, poverty associated with overcrowded housing [36,40] extreme temperature, hot indoor air, air quality concerns, smoking, mould [35,39,50] inadequate space for movement [38,43] lack of workstations, lack of free space to create one [39,43] need for workspace cleaning > still wet after cleaning [43] pets, animal hair [[34], [35], [36], [37], [38],^43^,^50^,^51^] poor lighting [34,35,37,43,59]	Require removal of pets from the room before care [37,38] Require removal of items of potential risk to staff [38] Clear space for medical supplies [37] Hang thing on door knob or around neck [36]
Tools	absence of complete work clothing [40,43] variation in patients' insurance coverage of equipment [36] no re-financing of IPC equipment and time for IPC [46] lack of information, lack of transfer of information [30,43,46] reliance on the quality of the information [36]	guidance on how to address contaminated households [43] proper assessment of home environment [51] tool to report safety concerns [31] plan nurse's schedule according to household cleanliness/risk [36,43] flagging MDRO colonization, color-coding infection type in the patient chart [54]
Tasks	high exposure activities e.g. bathing a patient [31] working alone with no physical support [31] intermittent nature of care [51]	Properly clean bed-bound/incontinent patients [36]
**Hand hygiene**	**Barriers**	**Facilitators**
People • HHC professionals • Patients • Others	focus on hand hygiene leading to over-reliance on hand-sanitizers vs hand-washing [51] reservations on whether 5 HH moments fit the HHC environment [43] more HH opportunities leading to low adherence [58] patients not consenting to use their facilities [49]	Require provision of soap and towels by patients [38,48]
Environment • Physical • Socio-organizational	dirty patient environment leading to more HH opportunities [59] no running water or working sink [34,36,51]	
Tools	alcohol rubs kept in the car/not taken to the home [48] risk of losing the bottle if taken to the home [48] various ways of drying hands, including uniforms, used home towels [43,48]	Keep dedicated disinfectant in the home [40] Use the one paper towel in the dressing pack, alcohol-based handwipes [38,49] Provide a hand hygiene-kit to be used in unusual circumstances [38]
Tasks	trade-off between HH and monitoring patient to avoid falls [30]	
**PPE**	**Barriers**	**Facilitators**
People • HHC professionals • Patients • Others	lack of training on how to use PPE [31] higher education [32] low perceived efficacy [32] wearing glasses [53] allergy to gloves [49] difficulty communicating with patients, patients with hearing impairment [30,32] conflict with patient satisfaction [30,49]	Not knowing the patient [45] Awareness regarding role of PPE [45] Strong safety climate of the agency [55]
Environment • Physical • Socio-organizational	uncomfortable, heat, fogging, difficulty breathing/seeing [31,32,39]	
Tools	no or limited provision by the agency [30,31,45,53] need to bring PPE to the home [45,53] absence of gloves box and aprons in the home [40,43] poor dexterity and fit of gloves [49] extra expenses out of own pocket [30]	Deliver PPE to the home [40,43] Keep dedicated gloves in the home [40]
Tasks	not considered a risky procedure [32,53] PPE use not in accordance with agency policy for specific procedure [53]	Sufficient time for procedures necessitating PPE [53]
**Sharps injury/ blood/body fluid exposure prevention**	**Barriers**	**Facilitators**
People • HHC professionals • Patients • Others	poor disposal technique [39] recapping [39,40,49,50] time pressure, more time needed to use the safety device [39,57] not considered a risky procedure [44] patients leaving sharps around the house, unshielded, multiple use [39,41,42] patient moving, uncooperative, difficulty communicating [39,47] patient needs physical support, awkward postures [39,47] no disposal of full container by the client [38] distraction from others [39,47,49]	Patient education, instruction on how to use and dispose improvised sharp containers [39,42] Require provision of containers by patients [39] Staff education: group interactive trainings, communication tools [33] Education of staff on proper container use; place it upright in the nursing bag [42] Work experience “protective” [56] Strong safety climate of the agency, promotion of prevention plans, ensure work practice in line with plans [42,55]
Environment • Physical • Socio-organisational	lack of work space, equipment difficult to reach [39,47] clutter [39,47] poor lighting [39] disposal into household garbage, municipal waste collection sites, flushing down the toilet [40,42,43]	Set up clean safe work area, free of distractions [39]
Tools	lack of sharps containers, overfilled containers, poor container design [42,49] unsafe improvised containers e.g. soft plastic bottles [33] no provision of safety device by the agency, having to spend own money [33,44] safety device not available at the visit [44] sharps from different providers in the same home, agency-provided versus patient-procured, patient-procured sharps often without safety device [42] safety device malfunctioning/ineffective, varying types [39]	Provision of containers and safety devices by the agency [39,43,44] Have two containers ready [39] Improved container design, leakproof cover [39] Improved safety feature design: easy to use, standardization, improve retractable design [39,42,50] Improvised containers: puncture resistant, e.g. laundry detergent bottle [42] Reduce costs for safety devices [39,42] Prefer needleless system e.g. jet injectors [39,42] Sharp disposal options for patients: disposal kiosks, return-by-mail boxes, municipal collection sites, pharmaceutical companies to provide containers with medication [42]
Tasks	lack of container if one-off injection [38] transport in nursing bag [39] high exposure activities [39,42] performing extra tasks that are not in the job description [33] helping clients to use a sharp [41]	Greater assistance in patient care [50]
**Aseptic technique**	**Barriers**	**Facilitators**
People • HHC professionals • Patients • Others	uncertainty and ambivalence about the nature and value of aseptic technique [52] being confronted with different approaches [52]	
Environment • Physical • Socio-organisational	clutter, lack of workspace [17] difficulty separating clean and soiled materials [17]	
Tools	costs for wound dressing materials not covered [38]	Use of clean towel, cleanable pate, plastic surface, or surgical drape on the table [17,36,38,43]
Tasks	infusions: pre-preparation of syringe for flushing Port-à-Cath [40] urinary catheter management: flushing with tap water, syringe re-used [40]	
**Decontamination/ Reprocessing, Waste management**	**Barriers**	**Facilitators**
People • HHC professionals • Patients • Others	little attention paid to the nursing bag [43] extensive use of smartphones and tablets, rarely disinfected [43]	Leaving nursing bag outside the home, taking minimal supplies into the home [35,51,54]
Environment • Physical • Socio-organizational	inadequate storage space for products [43,38] materials dependent on a third party to deliver, and storage by the patient [38,43]	Store materials in the cupboard, tape opened materials, use sterile specimen containers [38]
Tools	re-use of single-use disposable materials and cleaning with hand disinfectant [43] re-usage of gloves by different nurses or on different patients [43] re-storage of opened materials [38] disposal in household garbage [40]	Use a barrier for nursing bag or supplies [51,58] Use of dedicated equipment for MDRO patients [54]
Tasks		

HH: hand hygiene; HHC: home healthcare; IP: infection prevention; IPC: infection prevention and control; MDRO: multi-drug resistant organisms; PPE: personal protective equipment.

Study findings about facilitators or mitigation strategies were more fragmentary and often non-specific (Table II). Noteworthy findings included the provision of a hand hygiene-kit to be used in unusual circumstances [38], planning of nurses' schedules according to the infection risk assessment [36,43], facilitating sharps disposal for patients by offering them various disposal options [42], and the use of dedicated equipment for MDRO-colonised patients [54]. Further facilitators reported were provision of guidance on how to address contaminated households [43], and individual agreements with patients [31], such as removal of pets from the room before care procedures [37,38].

## Discussion

### Summary of main results

This scoping review mapped 33 studies addressing barriers and facilitators to infection prevention practices in HHC. A large proportion of studies focused on sharps injury or blood exposure prevention, and the majority of studies were conducted in the US. Study designs were largely qualitative and observational, with only one interventional study identified. Content analysis of barriers revealed a sum of findings that can characterise the HHC context that is relevant to infection prevention implementation. Study findings about facilitators were more fragmentary.

### Limitations of this review

The generalisability of findings from the variety of study settings may be limited, despite having limited the review to high-/upper-middle-income countries. We observed, however, substantial code saturation during the data extraction process, with similar findings arising in studies from various countries (e.g., both European and US studies revealed uncertainty about cost coverage for IPC equipment, and a lack of hand-washing facilities) [34,36,43,46]. These observations may legitimate drawing on the findings for building a framework of challenges that may serve research and implementation work across different countries.

The conceptual boundaries of research questions in this field are ill-defined. Our search strategy omitted very specific search terms and may thus have missed studies focusing on specific tasks or patient populations (such as total parenteral nutrition or peritoneal dialysis).

Our descriptive statistics aimed to represent research activity based on publications addressing different research questions, and thus deliberately double-count study datasets that resulted in more than one publication. This approach leads to overrepresentation of three studies from the US [[35], [36], [37],^44^,^47^,^53^,[55], [56], [57], [58], [59]].

In line with the aims of a scoping review, we did not attempt to assess certainty in the study findings. The methodological approaches of the included studies suggest the level of evidence generated is low. Survey studies are especially prone to selection bias (as illustrated by often low response rates) and various information biases, such as recall bias or desirability bias. The many limitations of survey studies suggest that direct observation studies, which are however subject to Hawthorne bias, provide more accurate results and should be given more weight in future analyses. Furthermore, behavioural and environmental variables used in the studies (e.g. safety climate [55], environmental stressors [50]) lack standardisation and validation of the scales used.

### Recommendations for future research

This review demonstrates a dominant research interest in sharps injury/blood exposure prevention, legitimated by an occupational safety perspective focusing on transmission prevention of bloodborne infectious diseases. Current challenges in the healthcare sector from a patient safety perspective, however, involve the spread of MDRO [60], and a shift of complex medical care to the outpatient and home care sector [2]. This suggests implementation of aseptic technique and decontamination/reprocessing in the home care sector is under researched.

The methodological limitations of the included observational studies suggest there is need for more interventional studies to provide evidence for targeted implementation measures. Research into IPC implementation interventions is becoming established in other healthcare settings [61,62].

Furthermore, this review reveals reservations of HHC professionals on the usefulness and practicability of established IPC recommendations for their work setting. Research into what level of infection prevention is needed in the HHC context, and what adaptations of existing recommendations can possibly be made without putting patients and HCWs at risk, is lacking. The simplification of six steps of hand hygiene to a three-step procedure may serve as an example of successful de-implementation of unnecessary techniques in the field of IPC [63].

## Conclusion

The included studies generate a set of findings that characterise the specific HHC context relevant to infection prevention implementation. However, study designs are largely qualitative and observational, and arguably of low-quality evidence. Future research should include implementation intervention studies. Furthermore, implementation of aseptic technique and decontamination/reprocessing of equipment are poorly studied in the HHC setting and deserve more research interest considering the current trends and challenges in the healthcare sector.

## Conflict of interest statement

None of the authors declares any conflict of interest related to this research.

## Funding statement

No specific funding existed for this research project. NS and LB receive their salary through a grant of the 10.13039/100000001Swiss National Science Foundation obtained by NDL (PCEFP3_181355).

## Consent for publication

All listed authors revised and approved the final manuscript.

## Author contributions

LB: conceptualization, data curation, formal analysis, methodology, project administration, visualization, writing – original draft preparation; NS: data curation, formal analysis; NDL: methodology, supervision, validation, writing – reviewing and editing.

## Availability of data and materials

All relevant data analyzed are included in the manuscript or appendices.

## References

[1] The 2018 Ageing Report (2018).

[2] Genet N., Boerma W., Kroneman M., Hutchinson A., Saltman R.B. (2012). Home Care across Europe - Current structure and future challenges.

[3] (2020). Europe home care market size, share & trends analysis report by component, by region, and segment forecasts, 2020 - 2027. Grand View research.

[4] Möckli N., Simon M., Meyer-Massetti C., Pihet S., Fischer R., Wächter M. (2021). Factors associated with homecare coordination and quality of care: a research protocol for a national multi-center cross-sectional study. BMC Health Serv Res.

[5] Busnel C., Vallet F., Ludwig C. (2021). Tooling nurses to assess complexity in routine home care practice: Derivation of a complexity index from the interRAI-HC. Nurs Open.

[6] Technology Enabled Care Services (TECS). https://www.england.nhs.uk/tecs/ (accessed Jun. 06, 2023).

[7] Conley J., Snyder G.D., Whitehead D., Levine D.M. (2022). Technology-enabled Hospital at Home: Innovation for Acute Care at Home. NEJM Catal Innov Care Deliv.

[8] Shang J., Dick A., Larson E., Stone P. (2018). A research agenda for infection prevention in home healthcare. Am J Infect Control.

[9] Hoxha A., Duysburgh E., Mortgat L. (2021). Healthcare-associated infections in home healthcare: an extensive assessment, 2019. Euro Surveill.

[10] Fischer T. (2023). Home care in Germany during the COVID-19 pandemic: A neglected population?. J Nurs Scholarsh.

[11] Rowe T.A., Patel M., Conor R.O., Mcmackin S., Hoak V., Lindquist L.A. (2020). COVID-19 exposures and infection control among homecare agencies. Arch Gerontol Geriatr.

[12] Shang J., Ma C., Poghosyan L., Dowding D., Stone P. (2014). The prevalence of infections and patient risk factors in home health care: A systematic review. Am J Infect Control.

[13] (2022). Standard precautions for the prevention and control of infections.

[14] (2018). Infection prevention and control assessment framework at the facility level.

[15] (2019). Minimum requirements for infection prevention and control programmes.

[16] Mcdonald M.V., Brickner C., Russell D., Dowding D., Larson E.L. (2021). Observation of Hand Hygiene Practices in Home Health Care. J Am Med Dir Assoc.

[17] Steffens E., Spriet I., Van Eldere J., Schuermans A. (2019). Compliance with evidence-based guidelines for the prevention of central line-associated bloodstream infections in a Belgian home care setting: An observational study. Am J Infect Control.

[18] Chou D.T.S., Achan P., Ramachandran M. (2012). The World Health Organization ‘5 moments of hand hygiene’: the scientific foundation. J Bone Joint Surg Br.

[19] O’Grady N.P., Alexander M., Burns L.A., Dellinger E.P., Garland J., Heard S.O. (2011). Healthcare Infection Control Practices Advisory Committee (HICPAC). Guidelines for the prevention of intravascular catheter-related infections. Clin Infect Dis.

[20] Rowley S., Clare S. (2022). Is ANTT Achievable in the Home Healthcare Setting?. Home Healthc Now.

[21] Peters M., Godfrey C., McInerney P., Munn Z., Tricco A.C., Khalil H., Aromataris E., Munn Z. (2020). JBI Manual for Evidence Synthesis, JBI.

[22] Tricco A.C., Lillie E., Zarin W., O'Brien K.K., Colquhoun H., Levac etal D. (2018). PRISMA Extension for Scoping Reviews (PRISMA-ScR): Checklist and Explanation. Ann Intern Med.

[23] PRISMA 2020 flow diagram for new systematic reviews which included searches of databases and registers only. Available online: https://prisma-statement.org/prismastatement/flowdiagram.aspx) (accessed Feb. 02, 2023).

[24] L. Brockhaus, N. Sass, N.D. Labhardt. Barriers and facilitators to infection prevention practices in home healthcare: protocol for a scoping review of the qualitative and quantitative evidence. OSF Registries. Available online: https://osf.io/fbpyq.

[25] Systematic Review Accelerator. Available online: https://sr-accelerator.com/#/polyglot (accessed Feb. 02, 2023).

[26] World Bank country and lending groups (2023). https://datahelpdesk.worldbank.org/knowledgebase/articles/906519-world-bank-country-and-lending-groups.

[27] Peters M.D.J., Marnie C., Tricco A.C., Pollock D., Munn Z., Alexander L. (2020 Oct). Updated methodological guidance for the conduct of scoping reviews. JBI Evid Synth.

[28] Pollock D., Peters M.D.J., Khalil H., McInerney P., Alexander L., Tricco A.C. (2023 Mar 1). Recommendations for the extraction, analysis, and presentation of results in scoping reviews. JBI Evid Synth.

[29] Holden R.J., Carayon P. (2021 Nov). SEIPS 101 and seven simple SEIPS tools. BMJ Qual Saf.

[30] Osakwe Z.T., Osborne J.C., Samuel T., Bianco G., Céspedes A., Odlum M. (2021). All alone: A qualitative study of home health aides' experiences during the COVID-19 pandemic in New York. Am J Infect Control.

[31] Osei-Poku G.K., Szczerepa O., Potter A.A., Malone M.E., Fain B.A., Prentice J.C. (2021). Safety Trade-Offs in Home Care During COVID-19: A Mixed Methods Study Capturing the Perspective of Frontline Workers. Patient Safety.

[32] King E.C., Zagrodney K.A.P., McKay S.M., Hung V., Holness L.D. (2023). Determinants of nurse’s and personal support worker’s adherence to facial protective equipment in a community setting during the COVID-19 pandemic: A pilot study. Am J Infect Control.

[33] Amuwo S., Lipscomb J., McPhaul K., Sokas R.K. (2013). Reducing occupational risk for blood and body fluid exposure among home care aides: an intervention effectiveness study. Home Health Care Serv Q.

[34] Adams V., Song J., Shang J., McDonald M., Dowding D., Ojo M. (2021 Jun). Infection prevention and control practices in the home environment: Examining enablers and barriers to adherence among home health care nurses. Am J Infect Control.

[35] Russell D., Dowding D., Trifilio M., McDonald M.V., Song J., Adams V. (2021 May). Individual, social, and environmental factors for infection risk among home healthcare patients: A multi-method study. Health Soc Care Community.

[36] Dowding D., Russell D., Trifilio M., McDonald M.V., Shang J. (2020). Home care nurses’ identification of patients at risk of infection and their risk mitigation strategies: A qualitative interview study. Int J Nurs Stud.

[37] Sitzman K.L., Leiss J.K. (2009 Oct). Documentation of incidental factors affecting the home healthcare work environment. Home Healthc Nurse.

[38] Felemban O., St John W., Shaban R. (2015). Infection prevention and control in home nursing: case study of four organisations in Australia. Br J Community Nurs.

[39] Markkanen P., Quinn M., Galligan C., Chalupka S., Davis L., Laramie A. (2007). There’s no place like home: a qualitative study of the working conditions of home health care providers. J Occup Environ Med.

[40] Popp W., Hilgenhoner M., Dogru-Wiegand S., Hansen D., Daniels-Haardt I. (2006). [Hygiene in home care. A study with home care providers]. Bundesgesundheitsblatt Gesundheitsforschung Gesundheitsschutz.

[41] Brouillette N.M., Quinn M.M., Kriebel D., Markkanen P.K., Galligan C.J., Sama S.R. (2017). Risk of sharps injuries among home care aides: Results of the Safe Home Care survey. Am J Infect Control.

[42] Markkanen P., Galligan C., Laramie A., Fisher J., Sama S., Quinn M. (2015). Understanding sharps injuries in home healthcare: The Safe Home Care qualitative methods study to identify pathways for injury prevention. BMC Publ Health.

[43] Wendt B., Huisman-de Waal G., Bakker-Jacobs A., Hautvast J.L.A., Huis A. (2022). Exploring infection prevention practices in home-based nursing care: A qualitative observational study. Int J Nurs Stud.

[44] Leiss J.K. (2010). Provision and use of safety-engineered medical devices among home care and hospice nurses in North Carolina. Am J Infect Control.

[45] Corrêa Cordeiro J.F., Pavinski Alves A., Gir E., Oliveira Miranda D., Marin da Silva Canini S.R. (2016 Jul/Sep). Use of personal protective equipment in a home care service. Cogitare Enferm.

[46] Adler A.C., Spegel H., Wilke J., Holler C., Herr C. (2012). [Strategies to prevent the transmission of multidrug-resistant pathogens and their practical implementation in outpatient care]. Gesundheitswesen.

[47] Kim H., Kriebel D., Quinn M.M., Davis L. (2010). The Snowman: A model of injuries and near-misses for the prevention of sharps injuries. Am J Ind Med.

[48] Felembam O., John W.S., Shaban R.Z. (2012 Mar). Hand hygiene practices of home visiting community nurses: perceptions, compliance, techniques, and contextual factors of practice using the World Health Organization’s ‘five moments for hand hygiene’. Home Healthc Nurse.

[49] Bennett G., Mansell I. (2004). Universal precautions: a survey of community nurses’ experience and practice. J Clin Nurs.

[50] Gershon R.R.M., Pearson J.M., Sherman M.F., Samar S.M., Canton A.N., Stone P.W. (2009). The prevalence and risk factors for percutaneous injuries in registered nurses in the home health care sector. Am J Infect Control.

[51] Pogorzelska-Maziarz M., Chastain A.M., Mangal S., Stone P.W. (2020). J. Shang. Home Health Staff Perspectives on Infection Prevention and Control: Implications for Coronavirus Disease 2019. J Am Med Dir Assoc.

[52] Hallett C.E. (2000). Infection control in wound care: a study of fatalism in community nursing. J Clin Nurs.

[53] Leiss J.K., Sitzman K.L. (2011). Provision and use of personal protective equipment among home care and hospice nurses in North Carolina. Am J Infect Control.

[54] Kenneley I. (2012). Infection control in home healthcare: an exploratory study of issues for patients and providers. Home Healthc Nurse.

[55] Leiss J.K. (2014). Safety climate and use of personal protective equipment and safety medical devices among home care and hospice nurses. Ind Health.

[56] Leiss J.K. (2012). Work experience, work environment, and blood exposure among home care and hospice nurses. Ind Health.

[57] Quinn M.M., Markkanen P.K., Galligan C.J., Kriebel D., Chalupka S.M., Kim H. (2009). Sharps injuries and other blood and body fluid exposures among home health care nurses and aides. Am J Public Health.

[58] Dowding D., McDonald M.V., Shang J. (2020 Dec 2). Implications of a US study on infection prevention and control in community settings in the UK. Br J Community Nurs.

[59] McDonald M.V., Brickner C., Russell D., Dowding D., Larson E.L., Trifilio M. (2021). Observation of Hand Hygiene Practices in Home Health Care. J Am Med Dir Assoc.

[60] (2023). Antimicrobial resistance surveillance in Europe 2023.

[61] Nyantakyi E., Caci L., Castro M., Schlaeppi C., Cook A., Albers B. (2022). Implementation of infection prevention and control for hospitalized neonates: A narrative review. Clin Microbiol Infect.

[62] Scheithauer S., Bickenbach J., Heisel H., Fehling P., Marx G., Lemmen S. (2018). Do WiFi-based hand hygiene dispenser systems increase hand hygiene compliance?. Am J Infect Control.

[63] Tschudin-Sutter S., Sepulcri D., Dangel M., Ulrich A., Frei R., Widmer A.F. (2019). Simplifying the World Health Organization Protocol: 3 Steps Versus 6 Steps for Performance of Hand Hygiene in a Cluster-randomized Trial. Clin Infect Dis.

[64] Amuwo S., Sokas R.K., McPhaul K., Lipscomb J. (2011). Occupational risk factors for blood and body fluid exposure among home care aides. Home Health Care Serv Q.

[65] Backinger C.L., Koustenis G.H. (1994). Analysis of needlestick injuries to health care workers providing home care. Am J Infect Control.

