# Supplementary material for: Barriers and facilitators to infection prevention practices in home healthcare: a scoping review and proposed implementation framework
Source: Infect Prev Pract. 2024 Jan 30;6(1):100342. doi: 10.1016/j.infpip.2024.100342 (PMC10864853; doi:10.1016/j.infpip.2024.100342)
Supplement: Multimedia component 1 [file mmc1.docx]

**MEDLINE (Ovid)**

((infection ADJ3 prevent* ).tw,kf. OR (transmission ADJ3 prevent* ).tw,kf. OR (infection ADJ3 control* ).tw,kf. OR (transmission ADJ3 control* ).tw,kf. OR "standard precaution*".tw,kf. OR "hand hygiene".tw,kf. OR "respiratory hygiene".tw,kf. OR "personal protective equipment*".tw,kf. OR "aseptic technique".tw,kf. OR (safe* ADJ3 injection* ).tw,kf. OR (sharp* ADJ3 injur* ).tw,kf. OR decontaminat*.tw,kf. OR (reprocess* ADJ5 equipment* ).tw,kf. OR (waste ADJ3 manag* ).tw,kf. OR (environment* ADJ3 clean* ).tw,kf. OR "Infection Control"/ OR "Universal Precautions"/ OR Decontamination/ OR "Hand Disinfection"/ OR "Hand Hygiene"/ OR "infectious disease transmission, patient-to-professional"/ OR "infectious disease transmission, professional-to-patient"/)

AND

("Home healthcare".tw,kf. OR "home health care".tw,kf. OR homecare.tw,kf. OR "home care".tw,kf. OR "care at home".tw,kf. OR "dom* care".tw,kf. OR "home medical care".tw,kf. OR "in-house care".tw,kf. OR "home$based care".tw,kf. OR (home ADJ nursing ).tw,kf. OR "nursing at home".tw,kf. OR exp "Community Health Nursing"/ OR exp "Home Care Services"/ OR "Home Nursing"/)

**EMBASE (Ovid)**

((infection ADJ3 prevent* ).tw,kf. OR (transmission ADJ3 prevent* ).tw,kf. OR (infection ADJ3 control* ).tw,kf. OR (transmission ADJ3 control* ).tw,kf. OR "standard precaution*".tw,kf. OR "hand hygiene".tw,kf. OR "respiratory hygiene".tw,kf. OR "personal protective equipment*".tw,kf. OR "aseptic technique".tw,kf. OR (safe* ADJ3 injection* ).tw,kf. OR (sharp* ADJ3 injur* ).tw,kf. OR decontaminat*.tw,kf. OR (reprocess* ADJ5 equipment* ).tw,kf. OR (waste ADJ3 manag* ).tw,kf. OR (environment* ADJ3 clean* ).tw,kf. OR Infection Control/ OR Universal Precautions/ OR skin decontamination/ OR antisepsis/ OR hand disinfection/ OR hand washing/ OR protective equipment OR sharps injury/ OR patient-to-professional transmission/ OR professional-to-patient transmission/)

AND

("Home healthcare".tw,kf. OR "home health care".tw,kf. OR homecare.tw,kf. OR "home care".tw,kf. OR "care at home".tw,kf. OR "dom* care".tw,kf. OR "home medical care".tw,kf. OR "in-house care".tw,kf. OR "home$based care".tw,kf. OR (home ADJ nursing ).tw,kf. OR "nursing at home".tw,kf. OR exp Community Health Nursing/ OR exp Home Care/)

**CINAHL (EBSCO)**

((TI infection OR AB infection OR SU infection) N3 (TI prevent* OR AB prevent* OR SU prevent*)) OR ((TI transmission OR AB transmission OR SU transmission) N3 (TI prevent* OR AB prevent* OR SU prevent*)) OR ((TI infection OR AB infection OR SU infection) N3 (TI control* OR AB control* OR SU control*)) OR ((TI transmission OR AB transmission OR SU transmission) N3 (TI control* OR AB control* OR SU control*)) OR (TI "standard precaution*" OR AB "standard precaution*" OR SU "standard precaution*") OR (TI "hand hygiene" OR AB "hand hygiene" OR SU "hand hygiene") OR (TI "respiratory hygiene" OR AB "respiratory hygiene" OR SU "respiratory hygiene") OR (TI "personal protective equipment*" OR AB "personal protective equipment*" OR SU "personal protective equipment*") OR (TI "aseptic technique" OR AB "aseptic technique" OR SU "aseptic technique") OR ((TI safe* OR AB safe* OR SU safe*) N3 (TI injection* OR AB injection* OR SU injection*)) OR ((TI sharp* OR AB sharp* OR SU sharp*) N3 (TI injur* OR AB injur* OR SU injur*)) OR (TI decontaminat* OR AB decontaminat* OR SU decontaminat*) OR ((TI reprocess* OR AB reprocess* OR SU reprocess*) N5 (TI equipment* OR AB equipment* OR SU equipment*)) OR ((TI waste OR AB waste OR SU waste) N3 (TI manag* OR AB manag* OR SU manag*)) OR ((TI environment* OR AB environment* OR SU environment*) N3 (TI clean* OR AB clean* OR SU clean*)) OR (MH "Infection Control") OR (MH "Universal Precautions") OR (MH Handwashing) OR (MH "Sterilization and Disinfection") OR (MH "Disease Transmission, Professional-to-Patient") OR (MH "Disease Transmission, Patient-to-Professional"))

AND

((TI "Home healthcare" OR AB "Home healthcare" OR SU "Home healthcare") OR (TI "home health care" OR AB "home health care" OR SU "home health care") OR (TI homecare OR AB homecare OR SU homecare) OR (TI "home care" OR AB "home care" OR SU "home care") OR (TI "care at home" OR AB "care at home" OR SU "care at home") OR (TI "dom* care" OR AB "dom* care" OR SU "dom* care") OR (TI "home medical care" OR AB "home medical care" OR SU "home medical care") OR (TI "in-house care" OR AB "in-house care" OR SU "in-house care") OR (TI "home?based care" OR AB "home?based care" OR SU "home?based care") OR ((TI home OR AB home OR SU home) W1 (TI nursing OR AB nursing OR SU nursing)) OR (TI "nursing at home" OR AB "nursing at home" OR SU "nursing at home") OR (MH "Community Health Nursing") OR (MH "Home Health Care") OR (MH "Home Nursing"))
