# Supplementary material for: Barriers and facilitators to infection prevention practices in home healthcare: a scoping review and proposed implementation framework
Source: Infect Prev Pract. 2024 Jan 30;6(1):100342. doi: 10.1016/j.infpip.2024.100342 (PMC10864853; doi:10.1016/j.infpip.2024.100342)
Supplement: Multimedia component 2 [file mmc2.docx]

**PRISMA 2020 flowchart of the study selection process:**

*Barriers and facilitators to infection prevention practices in home healthcare: a scoping review and proposed implementation framework*

**Identification of studies via databases**

Duplicate records removed:

(n = 1064)

Records identified from:

Embase (n = 1604)

CINAHL (n = 1410)

MEDLINE (n= 713)

**Identification**

Records undergoing abstract/title screening

(n = 2663)

Records excluded

(n = 2581)

Reports sought for retrieval

(n = 82)

Reports not retrieved

(n = 0)

**Screening**

Reports assessed for eligibility

(n = 82)

Reports excluded:

Wrong participants (n = 9)

Wrong concept (n = 27)

Wrong context (n = 7)

Wrong type of source (n = 5)

Other (n = 1)

Studies included in review

(n = 33)

**Included**
